# Supplementary material for: Mitochondrial dysfunction induces ALK5-SMAD2-mediated hypovascularization and arteriovenous malformations in mouse retinas
Source: Nat Commun. 2022 Dec 10;13:7637. doi: 10.1038/s41467-022-35262-w (PMC9741628; doi:10.1038/s41467-022-35262-w)
Supplement: Supplementary file 1 — Supplementary Information [file 41467_2022_35262_MOESM1_ESM.pdf]

SUPPLEMENTARY FIGURE AND LEGENDS

Supplementary Figure 1. Silencing of *Tfam*, *Cox10,* or *Trx2* did not induce significant cell death during the sprouting assay.

Supplementary Figure 2. Depletion of *Tfam*, *Cox*10 and *Trx*2 in retinal vessels.

Supplementary Figure 3. EC ROS generation is not associated with retarded vessel growth.

Supplementary Figure 4. *Tfam*^{ECKO}, *Cox10*^{ECKO} and *Trx2*^{ECKO} retinas exhibit microaneurysm at advanced ages.

Supplementary Figure 5. *Tfam*^{ECKO}, *Cox10*^{ECKO} and *Trx2*^{ECKO} retinas exhibit AVM at advanced ages.

Supplementary Figure 6. The scRNA-seq analyses from WT, *Tfam*^{ECKO}, *Cox10*^{ECKO} and *Trx2*^{ECKO} retinas.

Supplementary Figure 7. ALK5 inhibitor rescues mitochondrial dysfunction-impaired EC sprouting.

Supplementary Figure 8. ALK5 inhibitor rescues the retarded vessel growth in the mutant mice.

Supplementary Figure 9. ALK5 inhibitor rescues the retarded vascular malformation in the mutant mice.

Supplementary Figure 10. *Smad2* genetic deficiency rescues the vascular malformation in *Tfam*^{ECKO} mice.

Supplementary Figure 11. Uncut gels for Supplemental Figures.

Supplementary Table 1. List of PCR primers.

Supplementary Table 2. List of antibodies.

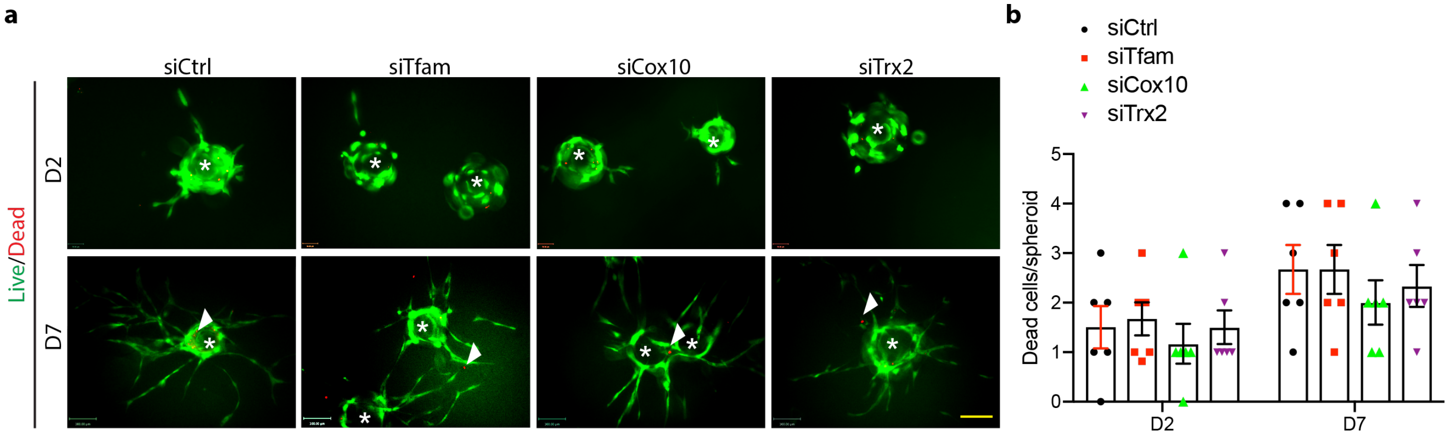

Supplementary Fig.1. **Silencing of *Tfam*, *Cox10,* or *Trx2* did not induce significant cell death during the sprouting assay.**

a-b. 48h after transfection with siRNA, HUVECs were coated with microbeads and grew in EGM2 medium as indicated days and stained with live/dead viability/cytotoxicity molecular probes. (a) Live cells were stained in green while dead cells were stained in red. Asterisks indicate beads and arrowheads for dead cells. (b) Quantification of dead cell number. 10 spheroids from each group were counted and three independent experiments were performed. Data are means ± SEM. *P* values are indicated, using one-way ANOVA followed by Tukey's multiple comparisons test. ns: non-significance (P>0.05). Scale bar: 50 μm. Source data are provided as a Source Data file.

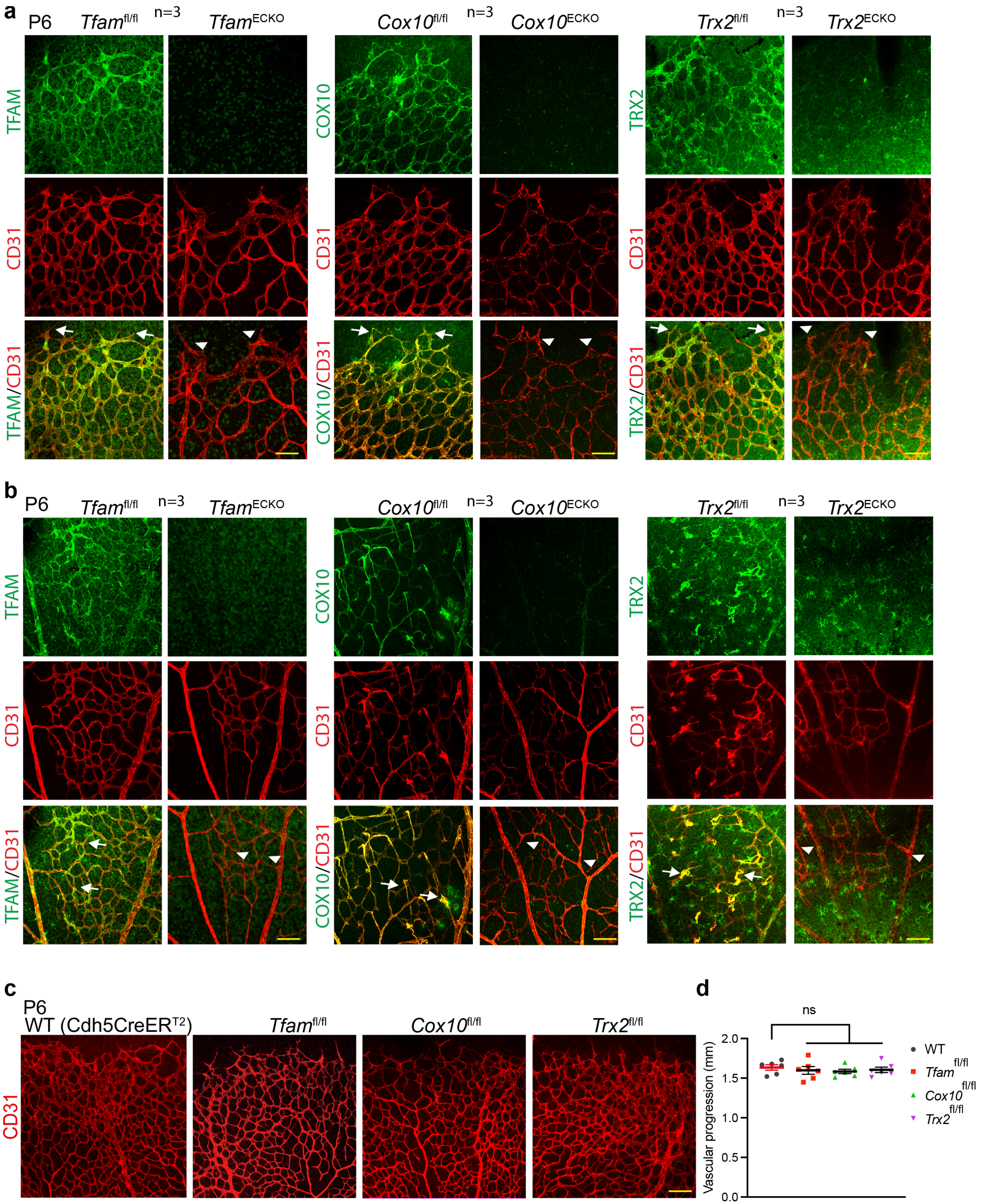

a
P6
Tfam fl/fl
n=3
Tfam ECKO
Cox10 fl/fl
n=3
Cox10 ECKO
Trx2 fl/fl
n=3
Trx2 ECKO
TFAM
CD31
TFAM/CD31
COX10
CD31
COX10/CD31
TRX2
CD31
TRX2/CD31
b
P6
Tfam fl/fl
n=3
Tfam ECKO
Cox10 fl/fl
n=3
Cox10 ECKO
Trx2 fl/fl
n=3
Trx2 ECKO
TFAM
CD31
TFAM/CD31
COX10
CD31
COX10/CD31
TRX2
CD31
TRX2/CD31
c
P6
WT (Cdh5CreERT2)
Tfam fl/fl
Cox10 fl/fl
Trx2 fl/fl
CD31
d
ns
Vascular progression (mm)
2.0
1.5
1.0
0.5
0.0
WT
Tfam fl/fl
Cox10 fl/fl
Trx2 fl/fl

Supplementary Fig.2. **Depletion of Tfam, Cox10 and Trx2 in retinal vessels.**

a-b. Paired littermates of *Tfam*$^{fl/fl}$ vs *Tfam*$^{fl/fl}$:*Cdh5*CreER^{T2}, *Cox10*$^{fl/fl}$ vs *Cox10*$^{fl/fl}$:*Cdh5*CreER^{T2}, *Trx2*$^{fl/fl}$ vs *Trx2*$^{fl/fl}$:*Cdh5*CreER^{T2} were feeded with tamoxifen at postnatal days (P) 1-3. The deletion efficiency of *Tfam*, *Cox10,* or *Trx2* in ECs was verified by whole-mount staining of the retinas with CD31 (red) together with TFAM, COX10 or TRX2 (green), respectively. Peripheral (A) and central areas (B) are shown. Arrows indicate positive vessels while arrowheads for negative vessels. n=3 mice for each strain. **c-d**. The floxed mice (*Tfam*$^{fl/fl}$, *Cox10*$^{fl/fl}$ and *Trx2*$^{fl/fl}$) showed similar vascular progression as WT (*Cdh5*CreER^{T2}) mice as visualized by whole mount staining with CD31 (c). Vascularized areas were quantified as a percentage of CD31 area/the total retinal surface (d). n=6 mice for each strain. Data are means ± SEM. ns: non-significance (P>0.05), using one-way ANOVA followed by Tukey's multiple comparisons test. Scale bar: 50 μm (a, b); 100 μm (c). Source data are provided as a Source Data file.

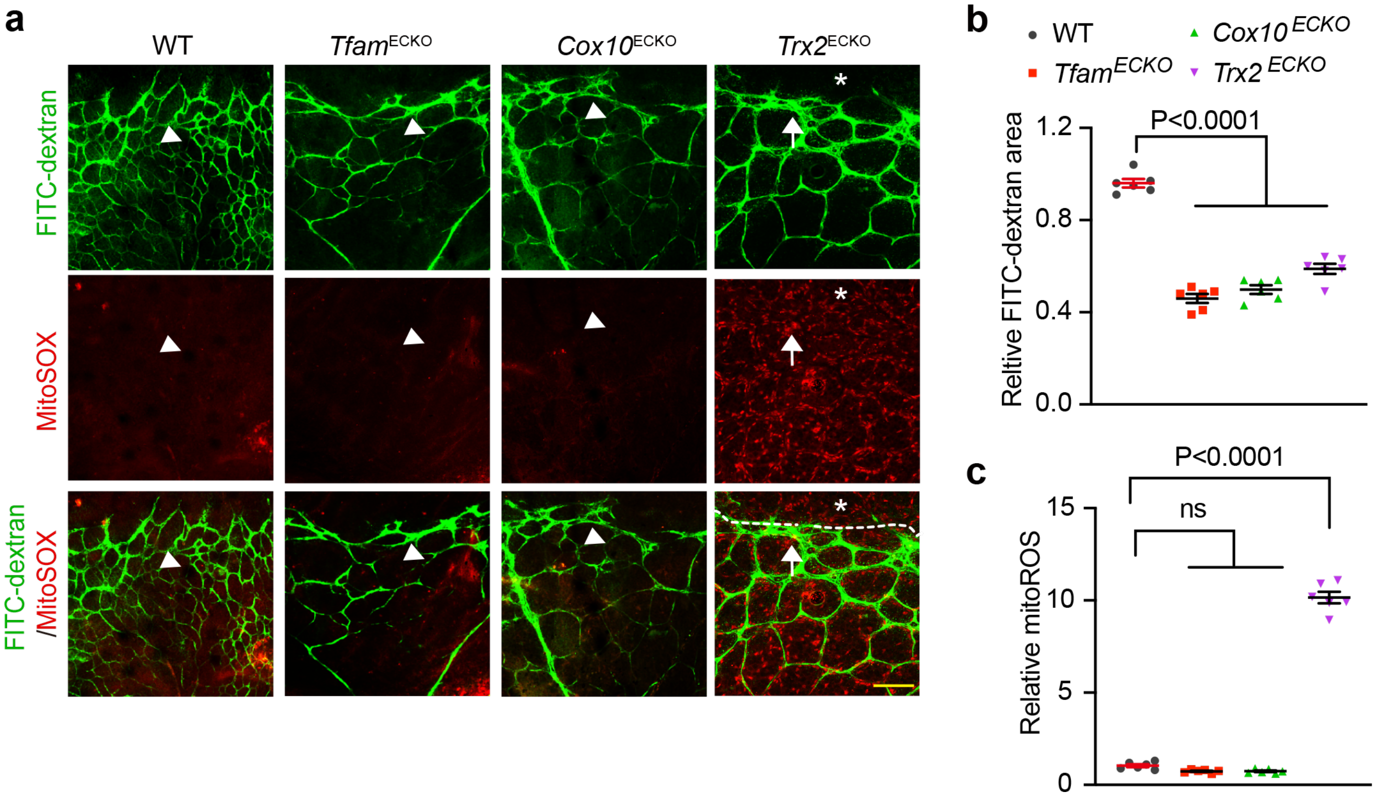

Supplementary Fig.3. EC ROS generation is not associated with retarded vessel growth.

a-c. P6 pups were injected retro-orbitally with FITC-dextran (2000 kDa) for 5 min and fresh retinas were subjected to MitoSox in situ staining (red). Arrowheads and arrows indicate mitoSOX$^-$ and mitoSOX$^+$ vascular areas, respectively. A mitoSOX$^+$ area outside the vasculature (separated with a dashed line) in *Trx2*^{ECKO} retina was indicated by an asterisk. Quantification of FITC-dextran$^+$ vascular areas (d) and mean fluorescence intensity (f). n=6 mice per group. Data are means ± SEM. *P* values are indicated, using one-way ANOVA followed by Tukey's multiple comparisons test. Scale bar: 25 μm (a). Source data are provided as a Source Data file.

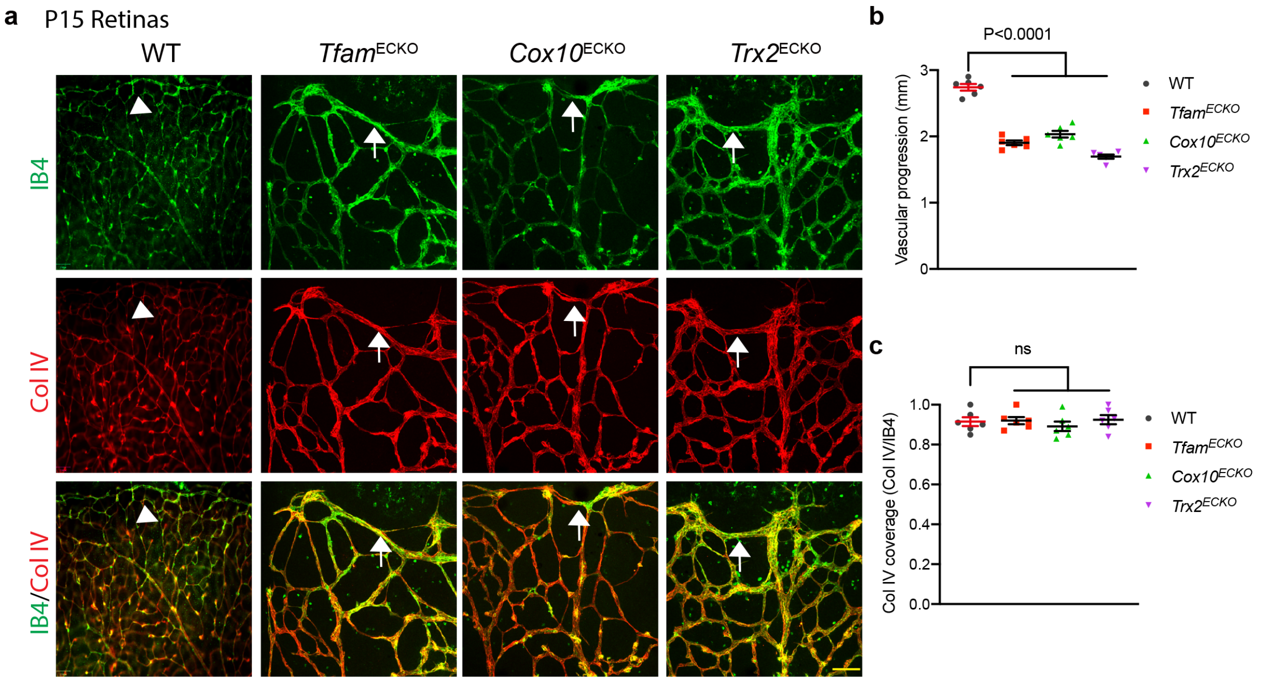

Supplementary Fig.4. *Tfam*^{ECKO}, *Cox10*^{ECKO} and *Trx2*^{ECKO} retinas exhibit microaneurysm at advanced ages. **a**. Visualization of collagen IV. P15 retinas were subjected to whole mount staining for IB4 with Col IV. Arrowhead and arrow indicate normal vessel in WT and AVM in the mutant retinas, respectively. **b-c**. Vascular progression (the distance of radial extension of the vascular plexus from the optic center) and Col IV coverage (Col IV/IB4 ratio) were quantified (b, c). n=6 mice per group. Data are means ± SEM. *P* values are indicated, using one-way ANOVA followed by Tukey's multiple comparisons test. ns: non-significance (P>0.05). Scale bar: 50 μm (a). Source data are provided as a Source Data file.

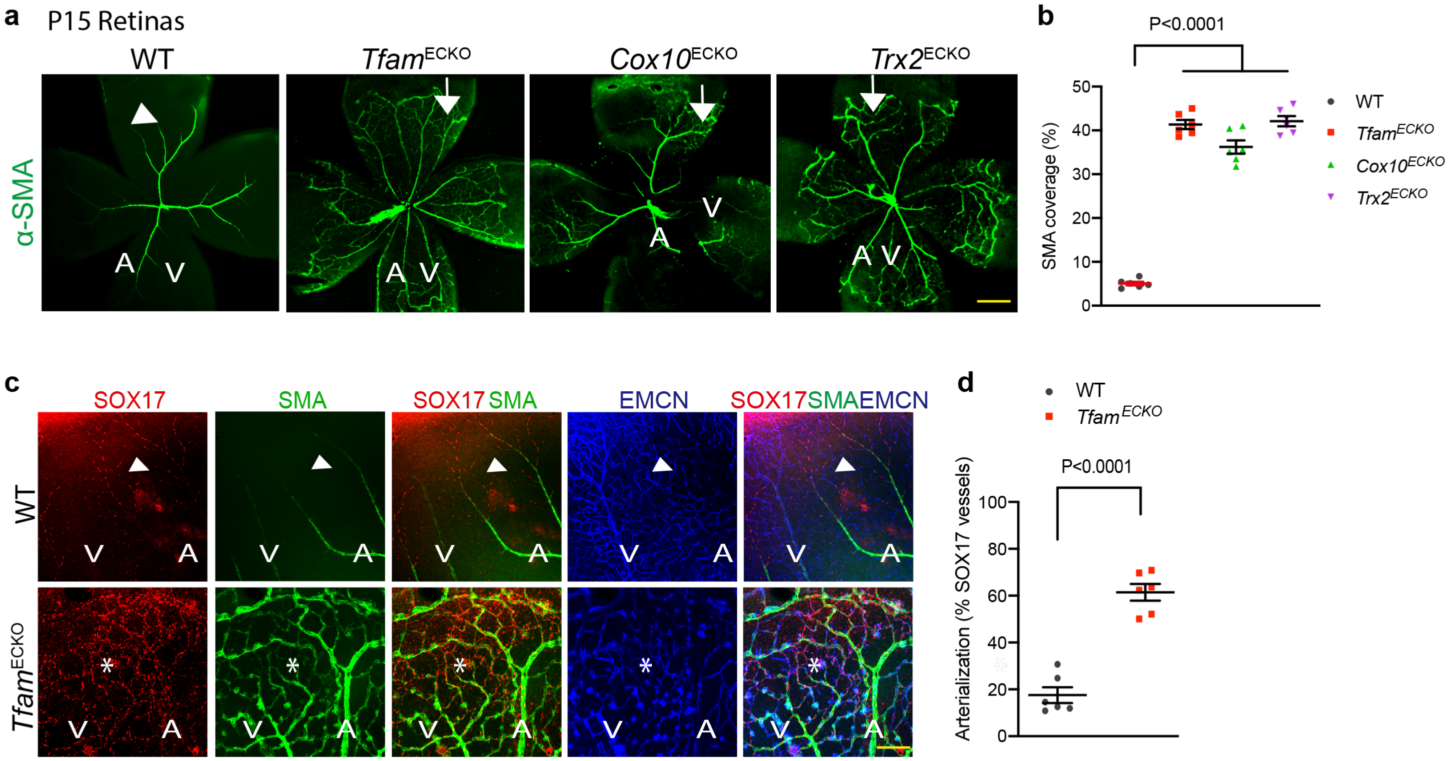

Supplementary Fig.5. *Tfam*^{ECKO}, *Cox10*^{ECKO} and *Trx2*^{ECKO} retinas exhibit arteriovenous malformation (AVM) at advanced ages. **a**. P15 retinas from WT, *Tfam*^{ECKO}, *Cox10*^{ECKO} and *Trx2*^{ECKO} were subjected to whole mount staining for α-SMA. Arrowhead and arrow indicate normal α-SMA^{-} microvessel area in WT and α-SMA^{+} microvessels in the mutant retinas, respectively. A: artery; V: vein. **b**. α-SMA area/retina area ratios in each group were quantified. n=6 mice per group. **c**. P15 retinas from WT and *Tfam*^{ECKO} were subjected to whole mount co-staining with SOX17, α-SMA and endomucin (EMCN). Arrowhead and arrow indicate normal SOX17^{-} microvessel area in WT and SOX17^{+} microvessels in the mutant retinas, respectively. A: artery; V: vein. **d**. SOX17^{+} microvessels in each group were quantified. n=6 mice per group. Data are means ± SEM. *P* values are indicated, using one-way ANOVA followed by Tukey's multiple comparisons test. Scale bar: 1 mm (a); 100 μm (c). Source data are provided as a Source Data file.

a

Sample	Estimated cell number	Median genes per cell	Reads mapped to genome	Median UMI counts per cell
WT	1,969	2,583	95.00%	6,938
Tfam^{ECKO}	897	2,549	94.50%	6,954
Cox10^{ECKO}	1,216	2,704	94.60%	8,007
Trx2^{ECKO}	1,105	1,722	94.80%	5,037

b

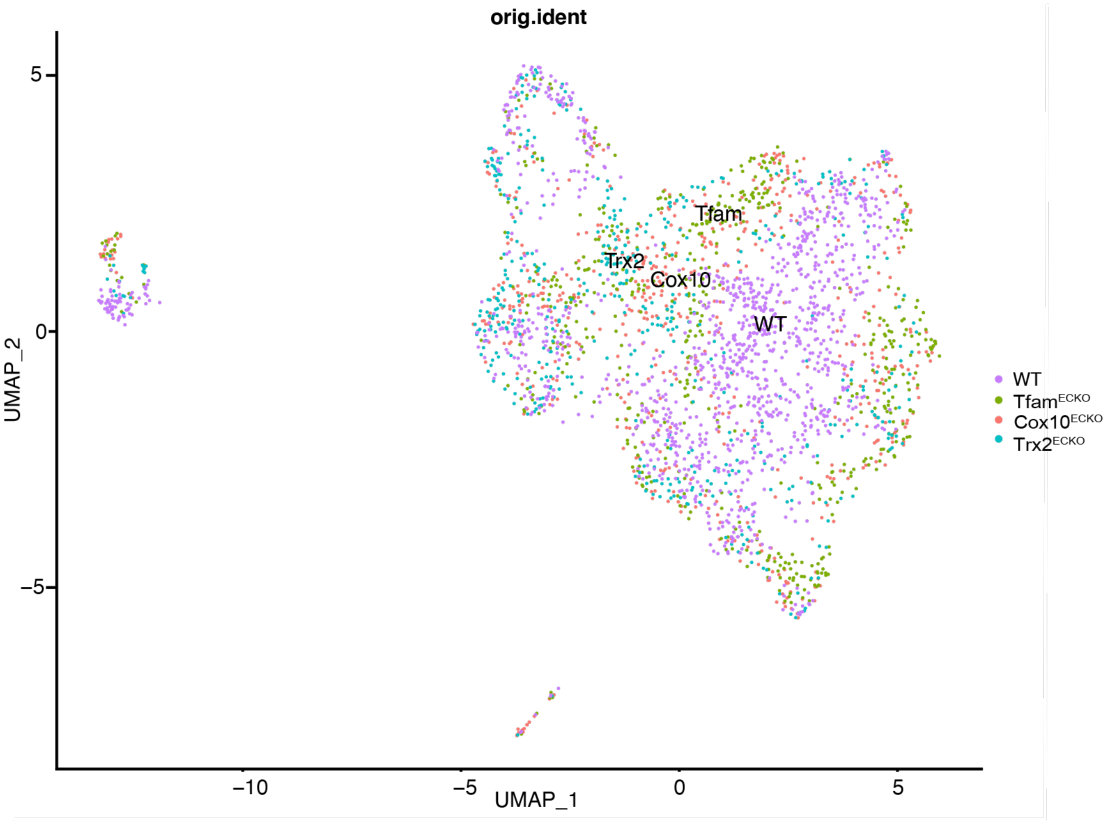

Supplementary Fig.6. The scRNA-seq analyses from WT, *Tfam*^{ECKO}, *Cox10*^{ECKO} and *Trx2*^{ECKO} retinas. a. Statistics of scRNA-seq in retinal ECs isolated from WT, *Tfam*^{ECKO}, *Cox10*^{ECKO} and *Trx2*^{ECKO} retinas. WT had more ECs collected compared to the mutant retinas but similar median genes per cell were obtained for 4 groups. **b.** UMAP projections for single-cell transcriptomes in ECs from WT, *Tfam*^{ECKO}, *Cox10*^{ECKO}, and *Trx2*^{ECKO} retinas colored accordingly to the sample of origin. Source data are provided as a Source Data file.

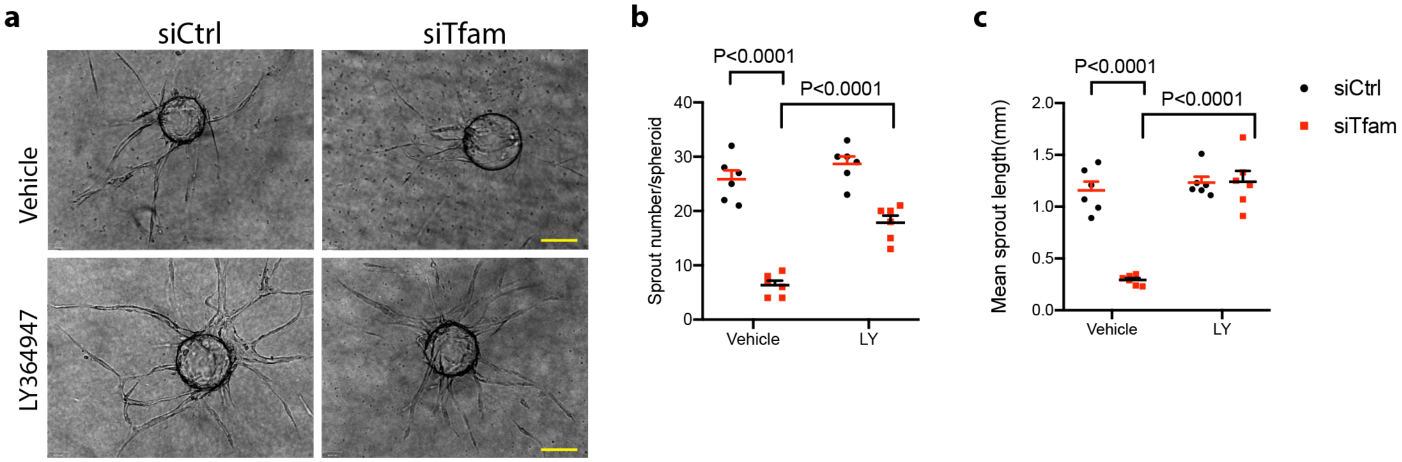

Supplementary Fig.7. ALK5 inhibitor rescues mitochondrial dysfunction-impaired EC sprouting in a 3D sprouting model. HUVECs were transfected with control or *Tfam* siRNAs. 48 h after transfection, cells were applied to spheroid sprouting assays in the absence or presence of ALK5 inhibitor LY364947 (5 μM). Imager were taken on day 7 (**a**), and quantification of the sprout number per spheroid (**b**) and mean sprout lengths (**c**) 10 spheroids per sample were counted. Data are duplicates from and three independent experiments. Data are means ± SEM. *P* values are indicated, using two-way ANOVA followed by Sidak's multiple comparisons test. Scale bar: 10 μm (a). Source data are provided as a Source Data file.

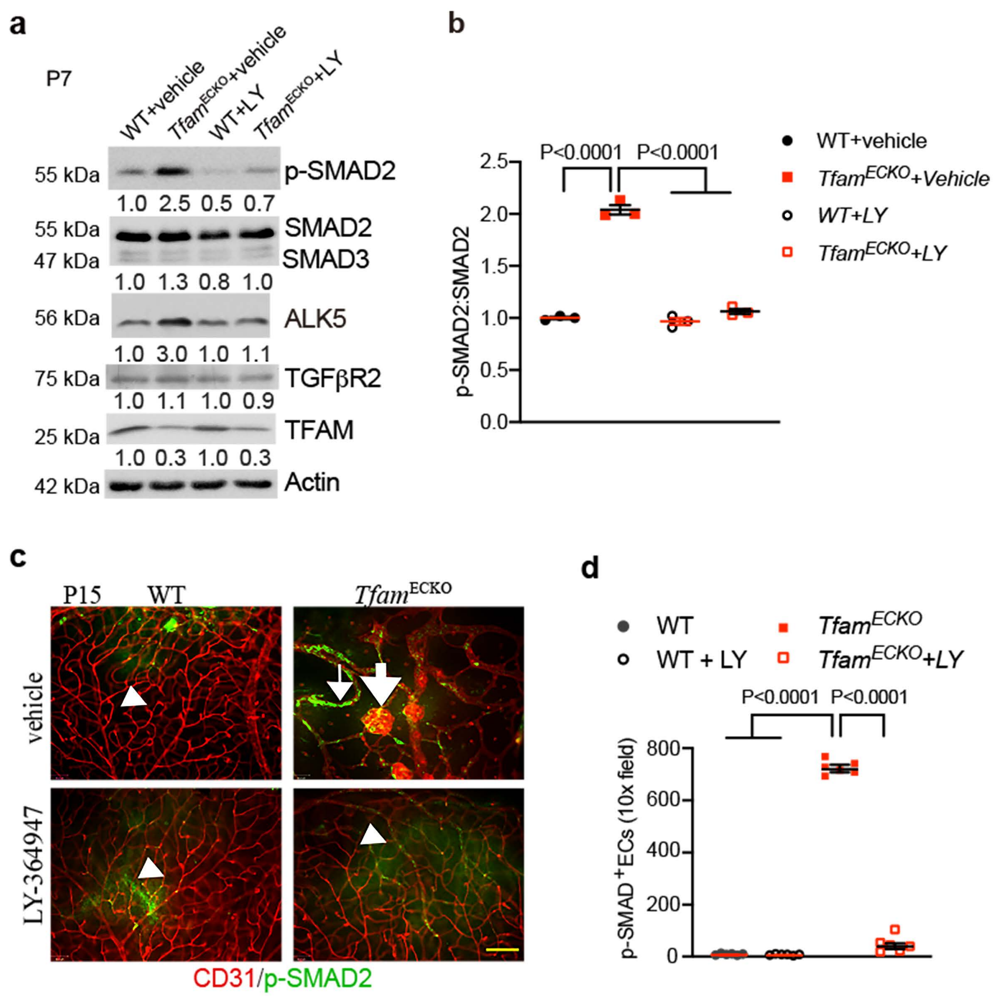

Supplementary Fig.8. ALK5 inhibitor attenuates EC proliferation and the retarded vessel growth in *Tfam*^ECKO^ retinas. WT and the mutant pups were received i.p. injection of vehicle or ALK5 inhibitor LY364947 compound at 5 μg/g body weight daily from P2-P6 or P2-P14. Mice were subjected to analyses at P7 or P15. **a-b**. Retina lysates were subjected to Western blotting with respective antibodies. Protein levels were quantified and presented as fold changes by vehicle WT as 1.0. Ratios of p-SMAD2:SMAD2 are presented as a graph. N= 3 mice per group. **c-d**. Visualization of p-SMAD2. P15 retinas were subjected to whole mount co-staining with p-SMAD2 and CD31. Arrowheads and arrows indicate normal $SMAD2^-$ vessels in WT and $SMAD2^+$ vessels in the mutant retinas, respectively. A large arrow indicates $SMAD2/3^+$ microaneurysm. Scale bar: 25 μm (c). Number of $SMAD2^+$ ECs (p-$SMAD2^+$ in CD31 vessels) were quantified. n=6 mice per group. Data in b and d are means ± SEM. *P* values are indicated, using one-way ANOVA followed by Tukey's multiple comparisons test. Source data are provided as a Source Data file.

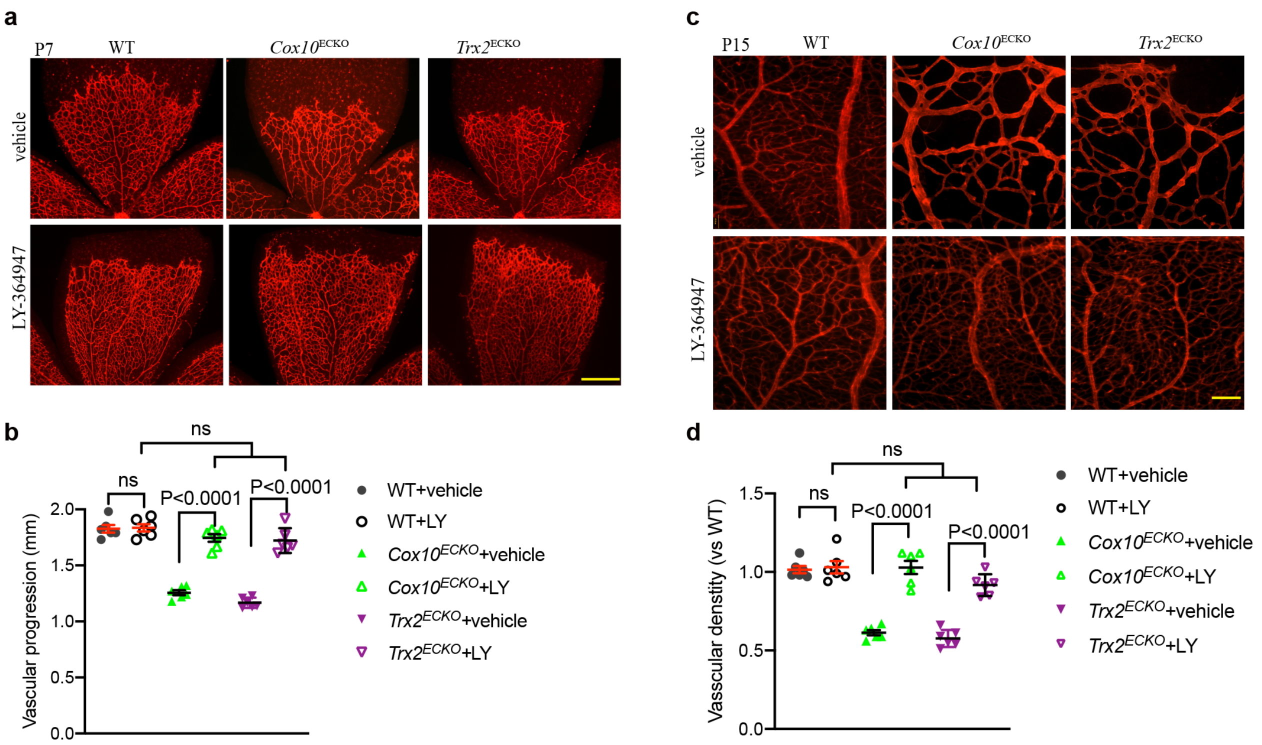

Supplementary Fig.9. **ALK5 inhibitor rescues the retarded vessel growth and vascular malformation in the mutant mice. a-b**. WT, *Cox10*^{ECKO} and *Trx2*^{ECKO} were injected with vehicle or ALK5 inhibitor LY364947 compound at 5 μg/g body weight daily i.p. from P2-P6 and retinas were harvested at P7 for CD31 whole-mount staining. Vascular progression (the distance of radial extension of the vascular plexus from the optic center) was quantified (b). n=6 mice per group. **c-d**. WT, *Cox10*^{ECKO} and *Trx2*^{ECKO} were injected with vehicle or ALK5 inhibitor LY364947 compound at 5 μg/g body weight daily i.p. from P2-P14 and retinas were harvested at P15 for CD31 whole-mount staining. Vessel density was quantified (d). n=6 mice per group. Data are means ± SEM. *P* values are indicated, using one-way ANOVA followed by Tukey's multiple comparisons test. ns: non-significance (P>0.05). Scale bar: 200 μm (b); 100 μm (c). Source data are provided as a Source Data file.

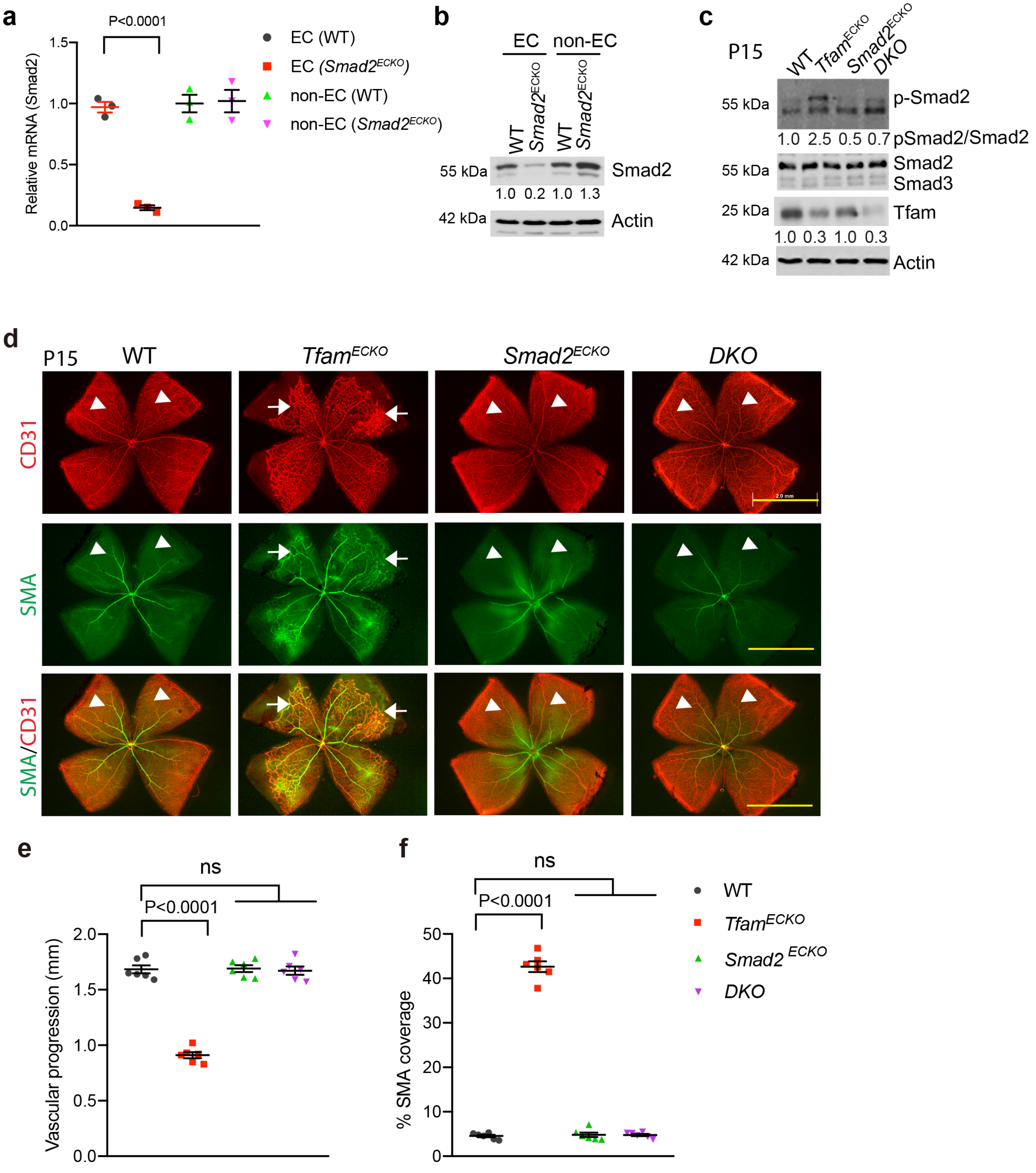

a
P<0.0001
Relative mRNA (Smad2)
1.5
1.0
0.5
0.0
EC (WT)
EC (Smad2ECKO)
non-EC (WT)
non-EC (Smad2ECKO)
b
EC
non-EC
WT
Smad2ECKO
WT
Smad2ECKO
55 kDa
Smad2
1.0 0.2 1.0 1.3
42 kDa
Actin
c
P15
WT
TfamECKO
Smad2ECKO
DKO
55 kDa
p-Smad2
1.0 2.5 0.5 0.7 pSmad2/Smad2
55 kDa
Smad2
Smad3
25 kDa
Tfam
1.0 0.3 1.0 0.3
42 kDa
Actin
d
P15
WT
TfamECKO
Smad2ECKO
DKO
CD31
SMA
SMA/CD31
e
ns
P<0.0001
Vascular progression (mm)
2.0
1.5
1.0
0.5
0.0
f
ns
P<0.0001
% SMA coverage
50
40
30
20
10
0
WT
TfamECKO
Smad2 ECKO
DKO

Supplementary Fig.10. ***Smad2* genetic deficiency rescues the vascular malformation in *Tfam*^{ECKO} mice**. **a-b**. *Smad2* deletion was specifically in mouse retinal ECs but not in non-EC cells. Mouse retinal ECs were isolated from P6 WT and *Smad2*^{ECKO} retinas by CellSorting as performed for scRNA-seq. (a) *Smad2* gene expression was determined by qRT-PCR. n = 3; ***P < 0.001 by unpaired two-tailed Student's t-test. (b) SMAD2 protein was determined by western blotting. Representative blot from three experiments. **c**. Retinal tissues from WT, *Tfam*^{ECKO}, *Smad2*^{ECKO} and DKO were harvested at P15. Phosphor- and total SMAD2 were determined by Western blotting. Representative blots were from three experiments. **d-f**. P15 retinas from WT, *Tfam*^{ECKO}, *Smad2*^{ECKO} and DKO were subjected to whole mount staining for α-SMA and CD31. Arrowheads indicate normal α-SMA$^-$ microvessel area in WT *Smad2*^{ECKO} and DKO retinas while arrows for α-SMA$^+$ microvessels in the *Tfam*^{ECKO} retinas, respectively. A: artery; V: vein. **(e)**. α-SMA area/retina area ratios in each group were quantified. **(f)**. Vascular progression (the distance of radial extension of the vascular plexus from the optic center) was quantified. n=6 mice per group. Data are means ± SEM. P values are indicated, using one-way ANOVA followed by Tukey's multiple comparisons test. ns: non-significance (P>0.05). Scale bar: 200 μm (d). Source data are provided as a Source Data file.

Supplementary Fig.11: Uncut gels

S8b

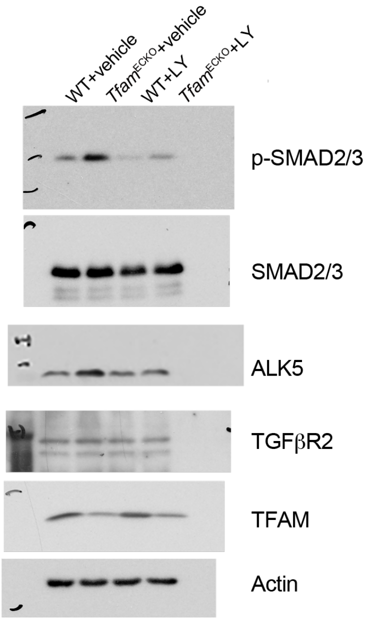

S10b

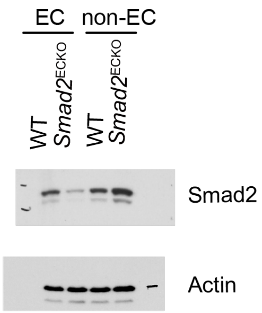

S10c

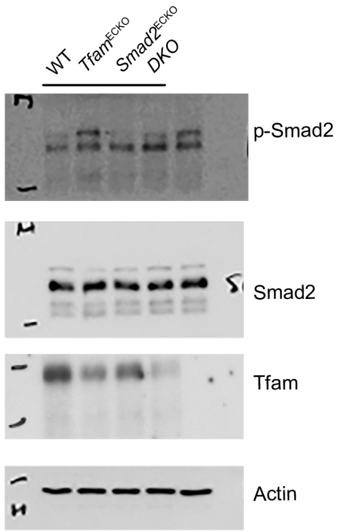

**Supplementary Table 1: Primers for genotyping, qRT-PCR or PCR:**		
Gene	Forward primer	Reverse primer
**Genotyping**		
Trx2	GGTGGCTCTATCCCACCTTG	GTAATTCCTGAACTCGGGAGAG
Cox10	GGCCTGCAGCTCAAAGTGTA	GAGAGGAGTCAAGGGGACCT
Tfam	CTCTAGCCCGGGTCCTATCT	TCCCTTGGGCCTTGTTTAC
qRT-PCR		
Trx2	CTGGTGGCCTGACTGTAACAC	TGACCACTCGGTCTTGAAAGT
Cox10	GCAAGTGTATGATTTGCCAGGA	TGCAGTGGTACTTACAACCAGA
Tfam	ATGGCGTTTCTCCGAAGCAT	TCCGCCCTATAAGCATCTTGA
18S rRNA	GTAACCCGTTGAACCCCATT	CCATCCAATCGGTAGTAGCG
Mitochondrial DNA copy		
mtDNA	GCCCCAGATATAGCATTCCC	GTTCATCCTGTTCCTGCTCC
Actb	ACCTTCTACAATGAGCTGCG	CTGGATGGCTACGTACATGG

**Supplementary Table 2: Antibodies for immunostaining**			
**Antibody name**	**Company**	**Cat #**	**Dilution**
CD31, rat	BD Pharmingen	553370	2 µg/mL
CD31, Armenian hamster	Millipore	MAB1398Z	2 µg/mL
Claudin-5, rabbit	Invitrogen	34-1600	2 µg/mL
Collagen IV, rabbit	Bio-Rad	2150-1470	2 µg/mL
Collagen IV, rabbit	AbD Serotec	2150-1470	2 µg/mL
COX10	Abcam	ab84053	2 µg/mL
Endomucin, rat	Hycult Biotech	HM1108	2 µg/mL
ERG, mouse	ThermoFisher	MA5-26245	1 µg/mL
ESM1 goat	ThermoFisher	BS-3615R	2 µg/mL
Fibronectin, rabbit	Abcam	Ab2413	2 µg/mL
Fibronectin, mouse	Abcam	Ab23750	2 µg/mL
Ki67	Cell Signaling	9027	2 µg/mL
NG2, rabbit	Millipore	AB5320	2 µg/mL
αSMA	Sigma	A2547	2 µg/mL
p-SMAD2/3, rabbit	ThermoFisher	PA5-36125	2 µg/mL
p-SMAD1/5/8, rabbit	Cell Signaling	9516	2 µg/mL
SMAD1	ThermoFisher	38-5400	2 µg/mL
SMAD2/3	Cell Signaling	3102	2 µg/mL
SMAD3	Abcam	Ab40854	2 µg/mL
TFAM, mouse	Santa Cruz	sc66695	8 µg/mL
TOMM20	Abcam	ab56783	2 µg/mL
TRX2, rabbit	Santa Cruz	Sc50336	8 µg/mL
VEGFR2, goat	R&D	AF644	2 µg/mL
VE-cadherin, rat	BD Pharmingen	555289	2 µg/mL
VE-cadherin, goat	Santa Cruz	sc-6458	2 µg/mL
ZO-1, rabbit	Invitrogen	61-7300	2 µg/mL
EdU assays using the Click-iT EdU kits	ThermoFisher	C10632	n/a
Alexa Flour 594 Isolectin GS-IB4	Invitrogen	I21413	1 µg/mL
Alexa Flour 488 Donkey Anti-Rat IgG	Invitrogen	A21208	1 µg/mL
Alexa Flour 488 Donkey Anti-Goat IgG	Invitrogen	A11055	1 µg/mL
Alexa Flour 488 Donkey Anti-Rabbit IgG	Invitrogen	A21206	1 µg/mL
Alexa Flour 488 Donkey Anti-Mouse IgG	Invitrogen	A21202	1 µg/mL
Alexa Flour 594 Donkey Anti-Rat IgG	Invitrogen	A21209	1 µg/mL
Alexa Flour 594 Donkey Anti-Goat IgG	Invitrogen	A11058	1 µg/mL
Alexa Flour 594 Donkey Anti-Rabbit IgG	Invitrogen	A21207	1 µg/mL
Alexa Flour 594 Donkey Anti-Mouse IgG	Invitrogen	A21203	1 µg/mL
