# Supplementary material for: Mitochondrial dysfunction induces ALK5-SMAD2-mediated hypovascularization and arteriovenous malformations in mouse retinas
Source: Nat Commun. 2022 Dec 10;13:7637. doi: 10.1038/s41467-022-35262-w (PMC9741628; doi:10.1038/s41467-022-35262-w)
Supplement: Supplementary file 3 — Reporting Summary [file 41467_2022_35262_MOESM3_ESM.pdf]

nature research

Corresponding author(s): Jenny Zhou and Wang Min

Last updated by author(s): 10/11/2022

Reporting Summary

Nature Research wishes to improve the reproducibility of the work that we publish. This form provides structure for consistency and transparency in reporting. For further information on Nature Research policies, see our Editorial Policies and the Editorial Policy Checklist.

Statistics

For all statistical analyses, confirm that the following items are present in the figure legend, table legend, main text, or Methods section.

n/a	Confirmed	
☐	☒	The exact sample size (n) for each experimental group/condition, given as a discrete number and unit of measurement
☐	☒	A statement on whether measurements were taken from distinct samples or whether the same sample was measured repeatedly
☐	☒	The statistical test(s) used AND whether they are one- or two-sided<br>*Only common tests should be described solely by name; describe more complex techniques in the Methods section.*
☒	☐	A description of all covariates tested
☐	☒	A description of any assumptions or corrections, such as tests of normality and adjustment for multiple comparisons
☐	☒	A full description of the statistical parameters including central tendency (e.g. means) or other basic estimates (e.g. regression coefficient) AND variation (e.g. standard deviation) or associated estimates of uncertainty (e.g. confidence intervals)
☐	☒	For null hypothesis testing, the test statistic (e.g. F, t, r) with confidence intervals, effect sizes, degrees of freedom and P value noted<br>*Give P values as exact values whenever suitable.*
☒	☐	For Bayesian analysis, information on the choice of priors and Markov chain Monte Carlo settings
☒	☐	For hierarchical and complex designs, identification of the appropriate level for tests and full reporting of outcomes
☒	☐	Estimates of effect sizes (e.g. Cohen's d, Pearson's r), indicating how they were calculated

Our web collection on statistics for biologists contains articles on many of the points above.

Software and code

Policy information about availability of computer code

Data collection	Axiovert 200 (Zeiss); Prism 6.0 and 8.0 software (GraphPad); Leica TCS SP8 STED; Morada CCD and iTEM (Olympus); FEI Tecnai TF20 at 200Kv;SerialEM3.8 (Boulder) on a FEI Eagle 4Kx4K CCD camera; confocal Leica SP5 microscope (Leica, Germany); confocal Zeiss Airyscan 880 microscope (Zeiss, Germany);iCycler real-time detection system (Bio-Rad Laboratories, Inc., Hercules, CA); NextSeq 6000 (Illumina, San Diego, CA)
Data analysis	NIH Image J 1.52P for imaging analyses; Prism8.0 software (GraphPad) for statistical analyses; Morada CCD and iTEM (Olympus) cellSens Dimension software; Imod4.9 (University of Colorado Boulder); Cell Ranger (v3.0.2; 10x Genomics); Seurat SCTransform function (v2); GOstats Bioconductor package (v2.46.0)

For manuscripts utilizing custom algorithms or software that are central to the research but not yet described in published literature, software must be made available to editors and reviewers. We strongly encourage code deposition in a community repository (e.g. GitHub). See the Nature Research guidelines for submitting code & software for further information.

Data

Policy information about availability of data

All manuscripts must include a data availability statement. This statement should provide the following information, where applicable:

- Accession codes, unique identifiers, or web links for publicly available datasets
- A list of figures that have associated raw data
- A description of any restrictions on data availability

All data, including data associated with main figures and supplementary figures, are available within the article or in the online-only Data Supplement or from the corresponding author on request. Source data are provided with this paper.
- Accession codes, unique identifiers, or web links for publicly available datasets- scRNA-seq data have been deposited and accession codes are provided. The

scRNA-seq data have been uploaded to the Gene Expression Omnibus (GEO) data repository (http://www.ncbi.nlm.nih.gov/projects/geo/) under accession number GSE172230.
- A list of figures that have associated raw data: Main Figures (Fig.1-10) and all Supplementary tables (Tables 1-5) and figures (Fig.1-11) are associated with raw data.
- A description of any restrictions on data availability: No any restrictions on data availability.

Field-specific reporting

Please select the one below that is the best fit for your research. If you are not sure, read the appropriate sections before making your selection.

[x] Life sciences [ ] Behavioural & social sciences [ ] Ecological, evolutionary & environmental sciences

For a reference copy of the document with all sections, see nature.com/documents/nr-reporting-summary-flat.pdf

Life sciences study design

All studies must disclose on these points even when the disclosure is negative.

Sample size	Sample sizes were chosen based on each experiment being technically feasible from a work flow standpoint while also providing a reasonable number of replicates to be confident in the results. For in vivo, group sizes were determined by an a priori power analysis for a two-sided, two-sample t-test with an α of 0.05 and power of 0.8 to detect a 10% difference in lesion size at the endpoint. For in vitro experiments, no sample-size calculations have been preformed in this study. At least n=3 biological triplicates were performed in all experiments unless explicitely indicated.
Data exclusions	No animals were discarded. No data were excluded from the data analyses.
Replication	All attempts at replication were successful
Randomization	Mice were randomly assigned to given treatment groups. For in vitro experiments, all comparison was conducted with appropriate controls, thus randomization was not necessary.
Blinding	For in vivo samples, the investigators were blinded to group allocation during data collection and analysis. For in vitro samples, this is not relevant because all values were determined without investigators' bias.

Reporting for specific materials, systems and methods

We require information from authors about some types of materials, experimental systems and methods used in many studies. Here, indicate whether each material, system or method listed is relevant to your study. If you are not sure if a list item applies to your research, read the appropriate section before selecting a response.

Materials & experimental systems

n/a	Involved in the study	
[ ]	[x]	Antibodies
[ ]	[x]	Eukaryotic cell lines
[x]	[ ]	Palaeontology and archaeology
[ ]	[x]	Animals and other organisms
[x]	[ ]	Human research participants
[x]	[ ]	Clinical data
[x]	[ ]	Dual use research of concern

Methods

n/a	Involved in the study	
[x]	[ ]	ChIP-seq
[x]	[ ]	Flow cytometry
[x]	[ ]	MRI-based neuroimaging

Antibodies

Antibodies used	Antibodies for Western blotting: Antibody against COX10 (ab84053), TRX2 (ab185544) from Abcam; p-Akt (rabbit, 9271), Akt (rabbit, 9272), p-SMAD1/5/9 (13820), p-SMAD2/3 (8828), SMAD2/3 (8685), SMAD1 (9743), SMAD2 (5339), TFAM (8076) were from Cell Signaling Technology; TGFR1 (E-AB-70130; recognizing human and mouse), and TGFR2 (E-AB-60379) were from Elabscience; ALK1 was from Origen (AP01172PU-N) and ALK5 was from R&D (AF3025-sp; recognizing human); β-actin (mouse, A1978) were from Sigma; p-SMAD2 (44-244G) and SMAD1 (38-5400) were from ThermoFisher. All primary antibodies were diluted 1:1000. Antibodies for immunostaining: CD31 (rat, BD Pharmingen, 55330); CD31 (Hamster, Millipore MAB1398Z); Alexa Fluour 594 or 488 conjugated secondary antibodies (Invitrogen); full list antibodies for immunostaining see Supplementary Table 3.
Validation	All antibodies used are commercially available and validated for Western blotting and immunostaining by the suppliers. Additional information on specificity and species cross-reactivity, with links to key publications can be found on the manufacturer's website. We also validated antibodies using IgG isotype as controls for parallel staining.

Eukaryotic cell lines

Policy information about cell lines

Cell line source(s)	Human umbilical vein EC (HUVEC) were from Yale Endothelial Cell Facility (Yale University). ECs were grown in Microvascular Endothelial Cell Growth Medium-2 MV (EGM2, Lonza).
Authentication	Cell lines were not authenticated.
Mycoplasma contamination	All cell lines were tested negative for mycoplasma contamination.
Commonly misidentified lines (See ICLAC register)	No commonly misidentified cell lines were used.

Animals and other organisms

Policy information about studies involving animals; ARRIVE guidelines recommended for reporting animal research

Laboratory animals	Tfamfl/fl and Cox10fl/fl mice were obtained from Dr. Navdeep Chandel, Northwestern University) and Dr. Carlos Moraes (University of Miami), respectively. Trx2fl/fl, Tfamfl/fl and Cox10fl/fl mice crossed with Cdh5-CreERT2 mice in which the Cre recombinase expression is driven by the Cdh5 promoter to generate mice with inducible endothelial cell (EC)-specific deletion of these three genes. For the in vivo tamoxifen-induced gene deletion, tamoxifen (Sigma, T5648) was diluted at 10 mg/ml in corn oil and fed mice at a dose of 50 μg once daily from postnatal day (P)1 to P3. For pups, littermates were randomly separated into vehicle (WT) and tamoxifen-fed (Tfam2ECKO, Cox10ECKO and Trx2ECKO groups such that the sex ratios of mice in both groups were equal. For genetic rescue by Smad2 deficiency, Cdh5CreERT2:Tfamfl/fl were bred with Cdh5CreERT2:SMAD2fl/fl (obtained from collaborator Dr. Anne Eichmann at Yale) to Cdh5CreERT2:Tfamfl/fl:Smad2fl/fl. Smad2ECKO and TfamECKOSmad2ECKO (DKO; inducible EC-deletion of Tfam and Smad2) were obtained upon feeding Cdh5CreERT2:Smad2fl/fl and Cdh5CreERT2:Tfamfl/fl:Smad2fl/fl pups, respectively, at P1 to P3 with tamoxifen. Mice were housed in the animal care facility of Yale University under standard pathogen-free conditions with a 12 h light/dark schedule and provided with food and water ad libitum, temperature was between 20 and 24 °C and relative humidity between 45 and 65 rH. For pups, littermates were randomly separated into vehicle (WT) and tamoxifen-fed (Tfam2ECKO, Cox10ECKO and Trx2ECKO groups such that the sex ratios of mice in both groups were equal. For adult mice, male and female animals were used in equal numbers for all experiments.
Wild animals	No wild animal were used in this study.
Field-collected samples	No field-collected samples were used in this study.
Ethics oversight	All animal procedures were performed under protocols approved by Yale University Institutional Animal Care and Use Committee.

Note that full information on the approval of the study protocol must also be provided in the manuscript.
