# Supplementary material for: Mitochondrial dysfunction induces ALK5-SMAD2-mediated hypovascularization and arteriovenous malformations in mouse retinas
Source: Nat Commun. 2022 Dec 10;13:7637. doi: 10.1038/s41467-022-35262-w (PMC9741628; doi:10.1038/s41467-022-35262-w)
Supplement: Supplementary file 4 — Description of Additional Supplementary Files [file 41467_2022_35262_MOESM4_ESM.pdf]

Title: Supplementary Data 1:
Description: Top 50 genes in each cluster. The positive differentially expressed genes for each cluster were compared to all other cells.

Title: Supplementary Data 2:
Description: Top 50 GO list. The seven major EC clusters were subjected to gene ontology (GO) analyses. **a**. Top 50 GO pathways in each cluster. **b**. The representative three GO pathways in each cluster.

Title: Supplementary Data 3:
Description: 138 common significantly DEGs. Unbiased comparisons of differential expressed genes (DEGs) between the different mutant mouse models and WT. Specifically, the total EC populations in clusters 0-7 were combined together upon removing pericyte and neuron clusters, and DEGs were compared between each mutant with the WT. Two-sided Wilcoxon Rank Sum test adjusted *p-values*<0.5*,* based on Bonferroni correction using the total number of genes, among all 3 comparisons.

Title: Supplementary Data 4:
Description: Antibodies for immunoblotting and immunostaining.

Title: Supplementary Data 5:

Description: Specific primers for qRT-PCR.
